# A systematic review of evidence for and against routine surveillance imaging after completing treatment for childhood extracranial solid tumors

**DOI:** 10.1002/cam4.3110

**Published:** 2020-05-19

**Authors:** Jessica E. Morgan, Ruth Walker, Melissa Harden, Robert S. Phillips

**Affiliations:** ^1^ Centre for Reviews and Dissemination University of York York UK; ^2^ Department of Paediatric Oncology Leeds Children's Hospital Leeds UK

**Keywords:** adolescent, diagnostic imaging, neoplasms, pediatrics, population surveillance

## Abstract

**Background:**

Regular off‐treatment imaging is often used to assess for recurrence of disease after childhood cancer treatment. It is unclear if this increases survival, or what burden surveillance places on patients, families, or health‐care services. This systematic review examines the impact of routine surveillance imaging after treatment of pediatric extracranial solid tumors.

**Methods:**

Collaborative patient and public involvement informed the design and interpretation of this work. Thirteen electronic databases, conference proceedings, and trial registries were searched alongside reference list checking and forward citation searching from 1990 onwards. Studies were screened and data were extracted by two researchers. Risk of bias was assessed using a modified ROBINS‐I tool. Relevant outcomes were overall survival, psychological distress indicators, number of imaging tests, cost‐effectiveness, and qualitative data regarding experiences of surveillance programs. PROSPERO (CRD42018103764).

**Results:**

Of 17 727 records identified, 55 studies of 10 207 patients were included. All studies used observational methods. Risk of bias for all except one study was moderate, serious, or critical. Data were too few to conduct meta‐analysis; however, narrative synthesis was performed. Surveillance strategies varied, and poorly reported, involving many scans and substantial radiation exposure (eg, neuroblastoma, median 133.5 mSv). For most diseases, surveillance imaging was not associated with increased overall survival, with the probable exception of Wilms tumor. No qualitative or psychological distress data were identified.

**Conclusions:**

At present, there is insufficient evidence to evaluate the effects of routine surveillance imaging on survival in most pediatric extracranial solid tumors. More high‐quality data are required, preferably through randomized controlled trials with well‐conducted qualitative elements.

## Graphical abstract

This systematic review examines the impact of routine surveillance imaging after treatment of pediatric extracranial solid tumors on overall survival, psychological distress indicators, number of imaging tests, cost‐effectiveness, and qualitative data regarding experiences of surveillance programs. At present, there is insufficient evidence to evaluate the effects of routine surveillance imaging on survival in most pediatric extracranial solid tumors.

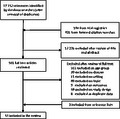

## 1 INTRODUCTION

Following completion of treatment for childhood malignancy, regular imaging studies are frequently used alongside clinical review to assess for recurrence of disease.^1^ It is anticipated that imaging may identify relapse before signs and symptoms develop, allowing earlier or less intensive treatment with an increased chance of survival.^2^

However, there has been some suggestion that this is not the case, as recurrence may still be detected clinically, and detection via imaging may not increase overall survival.^1^, ^3^, ^4^ Surveillance imaging comes with costs, including a psychological burden on families, possible increased risk of second malignancy, incidental findings, and increased exposure to general anesthesia, along with financial and opportunity costs to health services.^2^, ^4^

This systematic review seeks to establish if routine surveillance imaging after treatment of pediatric extracranial solid tumors causes more harm than good, specifically examining the impact on overall survival, anxiety and other psychological distress, number of scans received, radiation dose, other harms of surveillance, and cost‐effectiveness.

## 2 METHODS

The review protocol was prospectively registered on PROSPERO (CRD42018103764) and published.^5^

### 2.1 Patient and public involvement

A patient and public involvement (PPI) group steered the review from the outset, ranking important outcomes, understanding the context and implications of the results, including further research needs, and active involvement in the dissemination of the research. A young person with experience of childhood cancer summarized the review to be accessible to high‐school students (Supplemental File 1).

### 2.2 Searches

Electronic searches were undertaken from 1990 onwards, reflecting the current era of survival in childhood cancer. Published and unpublished studies were sought and no language or study design restrictions were applied, as such randomized controlled trials, quasi‐randomized studies, prospective, and retrospective cohort studies were all eligible to be included, as described in the protocol.^5^ The following databases were searched in July 2018: MEDLINE (Epub Ahead of Print, In‐Process & Other Non‐Indexed Citations, Ovid MEDLINE Daily, and Ovid MEDLINE), PubMed, EMBASE, PsycINFO, Cumulative Index to Nursing & Allied Health (CINAHL Plus), Science Citation Index, Conference Proceedings Citation Index—Science, Cochrane Database of Systematic Reviews (CDSR), Cochrane Central Register of Controlled Trials (CENTRAL), Health Technology Assessment (HTA) database, Database of Abstracts of Reviews of Effects (DARE), NHS Economic Evaluation Database (NHS EED), PROSPERO, and EconLit. (Supplemental File 2). Searches of conference proceedings of the RCPCH (Royal College of Paediatrics and Child Health), SIOP (International Society of Paediatric Oncology), ASPHO (American Society of Paediatric Hematology/Oncology), ASCO (American Society of Clinical Oncology), and ASH (American Society of Hematology), along with ClinicalTrials.gov and the WHO International Clinical Trials Registry Platform portal were undertaken. Reference lists of relevant systematic reviews and included articles were reviewed and forward citation searching of included articles was performed, using Web of Science.

### 2.3 Screening and data extraction

For inclusion and exclusion criteria see Box 1. Two authors independently screened the title and abstract of studies, dual‐screening 10% of the records and single‐screening the remaining 90% as agreement was good (96.6%). Disagreements were resolved by consensus, or by recourse to a third author. Data extraction was performed by two authors. Study quality was assessed using a modified ROBINS‐I tool, supplemented with potential sources of heterogeneity: patient demographic and clinical characteristics, study era, and geography.^6^, ^7^

Box 1 **Study inclusion and exclusion criteria**

Inclusion criteria:

‐ Population: Children or young people up to 25 years who had completed treatment for a malignant extracranial solid tumour and had no evidence of active and ongoing disease at end of treatment (or results for this subgroup)
‐ Intervention: programme of surveillance imaging aiming to detect relapse of previously treated childhood cancer
‐ Comparators: routine clinical review, another surveillance programme (using imaging or laboratory measures) or none (some studies reported this comparison as detection of relapses by surveillance compared to by symptoms)

Outcomes:

- Primary: Overall Survival (age at time of death or time from original diagnosis)
- Secondary: psychological distress indicators, number of imaging tests, cost‐effectiveness, qualitative data relating to experiences of surveillance imaging, other harms of imaging (as identified by the studies themselves)

Exclusion criteria

‐ Case studies
‐ Studies from Low and Middle‐Income Countries (LMIC) ‐ Only studies performed in high‐income countries were included, to reflect the treatment and surveillance strategies in these settings.
‐ Surveillance solely related to patients with cancer predisposition syndromes
‐ Surveillance looking predominantly for late effects of treatment

### 2.4 Analysis

Key study characteristics were summarized in narrative and tabular forms. Given the degree of clinical heterogeneity and absence of sufficient data, meta‐analysis was not appropriate. Narrative synthesis was performed by tumor type, and focused on the key themes of: method of identification of relapse, burden of surveillance programs, and effects on survival.

## 3 RESULTS

**Figure 1:**
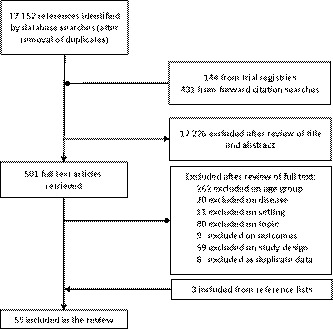
Flow diagram for study selection

About 17 727 unique records were identified by the search, 17 226 were excluded on title and abstract, and 449 excluded following full‐text review (Figure 1). Review of conference proceedings and references searches identified three further studies. Review of trial registries identified no ongoing relevant studies.

### 3.1 Mapping summary

Fifty‐five studies, with 10 423 participants, were included (Table 1). Most (48/55) studies were retrospective cohort studies. Twelve studies reported “mixed malignancies” where patients with more than one type of malignancy were included, including mixed lymphomas.^1^, ^8^, ^9^, ^10^, ^11^, ^12^, ^13^, ^14^, ^15^, ^16^, ^17^, ^18^ These studies do not provide sufficient granularity to advise clinical decision‐making and as such are not discussed further in this paper, information relating to these are provided in Supplemental File 3. Wilms tumor was the most frequently studied malignancy. Surveillance programs were poorly reported. Eight main imaging modalities were assessed (CT, gallium scan, ultrasound, X‐ray, MRI, FDG‐PT, bone scan, and MIBG), with most studies (32/55) assessing more than one modality and CT most commonly used (32/55 studies). In many studies, it was unclear whether surveillance imaging was within a clinical trial or part of routine care.

**Table 1:** Study characteristics

Study	Year of publication	Disease	Country	N participants	N participants experiencing relapse	Age (median^1^, mean^2^)	Details of surveillance program		
							Mode	Frequency	Maximum follow‐up period
Bayar^19^	2000	NHL	US	44	0	8.33^2^	CT, Gallium scans	3‐6 mo	6
Borst^20^	2013	NHL	US	12	0	12.42^2^	CT, Gallium scans, MRI, FDG‐PET, Bone scan	Every 3 mo for 12‐24 mo, decreasing with time	10
Eissa^21^	2014	NHL	US	44	3	8.88^2^	CT, Gallium scans, X‐ray, FDG‐PET	NR	10
Karantanis^22^	2010	NHL	US	10	0	36.1^2^	CT, FDG‐PET	NR	NR
Friedmann^23^	2013	Hodgkin's lymphoma	US	402	64	15.6^1^	CT, Gallium scans, X‐ray, FDG‐PET	Every 3 mo for 1 y, decreasing with time	18.3
Levine^24^	2006	Hodgkin's lymphoma	US	47	3	15^1^	CT, FDG‐PET	NR	5
Meany^25^	2007	Hodgkin's lymphoma	US	25	2	14.2^2^	CT, FDG‐PET	Every 3 mo for 2 y	3.83
Voss^26^	2012	Hodgkin's lymphoma	US	219	25	NR	CT, Gallium scans	NR	NR
Wickmann^27^	2003	Hodgkin's lymphoma	Germany	15	13	NR	CT, MRI, FDG‐PET	Every 6 mo, decreasing with time	NR
Chang^31^	2015	Osteosarcoma	South Korea	153	9	17^2^	FDG‐PET	Every 3‐6 mo for 2 y, decreasing with time	7.67
Korholz^28^	1998	Osteosarcoma	Germany	78	28	14^1^	CT, X‐ray, Bone scan, Other	Every 3‐6 mo for 1‐2 y, decreasing with time	15.11
Korholz^29^	1996	Osteosarcoma	Germany	78	28	14^1^	CT, X‐ray, Bone scan,	Monthly or bimonthly for 1‐2 y, decreasing with time	NR
Massera^32^	1994	Osteosarcoma	Italy	16	6	19^2^	CT	NR	NR
Korholz^30^	2000	Osteosarcoma & Ewing's sarcoma	Germany	119	7	15^1^	CT, X‐ray, Bone scan,	NR	14
Cash^33^	2013	Ewing's sarcoma	US	71	21	NR	NR	NR	NR
Heinemann^34^	2018	Ewing's sarcoma	Germany	180	30	13.8^1^	CT, X‐ray, MRI, FDG‐PET, Bone scan	Every 1.5 and 3 mo for 1 y, decreasing with time	5
Heinemann^35^	2017	Ewing's sarcoma	Germany	284	160	15^1^	CT, X‐ray, FDG‐PET, Bone scan	Every 1.5 and 3 mo for 1 y, decreasing with time	12
Brok^36^	2018	Wilms tumor	Brazil, France, Germany, Netherlands, UK	4271	538	4.22^1^	Ultrasound, X‐ray	Every 3 monthly for 12‐24 mo, decreasing with time	10
Carrico^37^	1997	Wilms tumor	US	60	7	3.5^2^	Ultrasound, X‐ray	Between 6 wks and 3 mo, variable on disease stage	5
Daw^38^	2002	Wilms tumor	US	280	8	30^1^	CT, Ultrasound	NR	NR
Kaste^39^	2013	Wilms tumor	US	110	16	2.92^1^	CT	NR	NR
Mullen^40^	2018	Wilms tumor	US, Canada, Australia, New Zealand, Switzerland, and Netherlands	336	281	NR	CT, Ultrasound, X‐ray	NR	NR
Davini^41^	2018	Hepatoblastoma	US	31	NR	NR	CT	NR	NR
Rojas^42^	2014	Hepatoblastoma	US	26	5	2.33^2^	CT, Ultrasound, MRI, FDG‐PET	Every 3 mo for 1 y, then at discretion of oncologist	NR
Bruggers^43^	1998	Neuroblastoma	US	32	22	3.75^2^	NR	NR	NR
Federico^44^	2015	Neuroblastoma	US	78	46	2.7^1^	CT, MIBG	NR	NR
Kushner^45^	2009	Neuroblastoma	US	154	154	NR	CT, MIBG	Every 2 to 4 months	NR
Okuyama^46^	2002	Neuroblastoma	Japan	40	5	1.2^1^	MIBG	Every 3‐12 mo, variable with disease stage	NR
Owens^47^	2016	Neuroblastoma	Canada	183	50	3.54^1^	MRI, MIBG	NR	NR
Cogswell^50^	1994	Rhabdomyosarcoma	Australia	40	10	5.83^1^	Gallium scans, X‐ray, Bone scan	Every 3 mo for 2 y, decreasing with time	NR
Lin^3^	2016	Rhabdomyosarcoma	US	145	24	13.5^2^	NR	NR	NR
Mallebranche^52^	2017	Rhabdomyosarcoma	France	99	NR	5^1^	Ultrasound, X‐ray	Every 2 mo for 2 y, decreasing with time	NR
Vaarwerk^51^	2018	Rhabdomyosarcoma	UK	182	182	NR	NR	NR	17.7
Sirin^48^	2016	Retinoblastoma	Germany	50	3	1.17^2^	MRI	NR	6
White^49^	1991	Retinoblastoma	Australia, US	15	0	21^2^	Bone scan	Every 4 mo, decreasing with time	5
Lobeck^53^	2017	Appendiceal carcinoid tumors	US	30	0	13.5^2^	CT, Ultrasound, MRI	NR	5.33
Geldart^54^	2006	Germ cell tumor	UK	329	20	23^1^	NR	NR	NR
Halalsheh^55^	2018	Malignant melanoma	US	21	4	14.0^1^	MRI/CT, PET/CT	Every 6 mo	
Cheuk^56^	2012	Nasopharyngeal carcinoma	US	18	1	13.6^1^	MRI, FDG‐PET	NR	NR
Zanetta^57^	1994	Ovarian cancer	Italy	106	9	NR	Ultrasound	Every 3 mo, decreasing with time	NR
Laddie^58^	2009	Testicular tumor	UK	51	12	NR	CT	NR	NR
Vali^59^	2015	Thyroid cancer	Canada	54	17	14.1^2^	Ultrasound	Every 6‐12 mo	10
Berrettini^60^	2015	Urothelial bladder neoplasms	UK	18	0	11^1^	Ultrasound	Varied per center	14.5

Abbreviations: NHL, Non‐Hodgkin’s lymphoma; NR, not reported; UK, United Kingdom; US, United States.

### 3.2 Risk of bias

Risk of bias was variable, with most studies demonstrating moderate to serious risk of bias (Table 2). Particular issues relate to confounding and lead‐time bias (where studies measure survival from time of detection of relapse, rather than from original diagnosis).

**Table 2:** Risk of bias for studies

Study	Year	Confounding	Patient selection		Protocol deviation		Missing data		Knowledge of intervention and recording of outcome		Effect estimate	Overall judgment of risk
Bayar^19^	2000											Moderate
Berrettini^60^	2015											Moderate
Borst^20^	2013											Moderate
Brok^36^	2018											Serious
Bruggers^43^	1998											Moderate
Carrico^37^	1997											Low
Cash^33^	2013											Moderate
Chang^31^	2015											Moderate
Cheuk^56^	2012											Moderate
Cogswell^50^	1994											Moderate
Davini^41^	2018											Moderate
Daw^38^	2002											Moderate
Eissa^21^	2014											Moderate
Federico^44^	2015											Moderate
Friedmann^23^	2013											Serious
Geldart^54^	2006											Serious
Halalsheh^55^	2018											Moderate
Heinemann^34^	2018											Moderate
Heinemann^35^	2017											Serious
Karantanis^22^	2010											Moderate
Kaste^39^	2013											Moderate
Korholz^28^	1998											Moderate
Korholz^29^	1996											Serious
Korholz^30^	2000											Serious
Kushner^45^	2009											Serious
Laddie^58^	2009											Moderate
Levine^24^	2006											Moderate
Lin^3^	2016											Moderate
Lobeck^53^	2017											Moderate
Mallebranche^52^	2017											Critical
Massera^32^	1994											Moderate
Meany^25^	2007											Moderate
Mullen^40^	2018											Moderate
Okuyama^46^	2002											Moderate
Owens^47^	2016											Moderate
Rojas^42^	2014											Moderate
Sirin^48^	2016											Moderate
Vaarwerk^51^	2018											Moderate
Vali^59^	2015											Moderate
Voss^26^	2012											Serious
Wickmann^27^	2003											Serious
Zanetta^57^	1994											Serious
White^49^	1991											Moderate

Note

Blue: no information; Green: low risk; Orange: moderate risk; Red: serious risk; Purple: critical risk.

### 3.3 Analysis

#### 3.3.1 Lymphomas

##### Non‐Hodgkin’s lymphoma

Four studies examined surveillance imaging in 110 patients with non‐Hodgkin’s lymphoma.^19^, ^20^, ^21^, ^22^ Data from three of four studies indicated large numbers of scans, with 806 scan conducted in 66 patients.^20^, ^21^, ^22^ Where reported, scanning was associated with notable radiation dose (median whole body radiation dose of 40.3‐91.3 MSv).^20^, ^21^

Three relapses occurred within one study population in a median time of 0.25 years. These were detected by symptoms.^21^ Surveillance imaging detected no relapse and produced 17 false positive images.^19^, ^20^, ^22^

##### Hodgkin’s lymphoma

Five studies assessed surveillance imaging in 799 patients with Hodgkin’s lymphoma.^23^, ^24^, ^25^, ^26^, ^27^ Surveillance programs comprised large numbers of images, where reported, 1293 in 291 patients.^24^, ^25^, ^26^

Relapse was detected in 111 (13.8%) patients, 51 (45.9%) by surveillance imaging and 60 (45.1%) by clinical signs and symptoms. Thirty‐four false positive images were reported in two studies.^24^, ^25^

One study reported a median time to relapse of 1.7 years by scan compared to 0.61 years in those detected by clinical signs and symptoms.^26^ For those relapses detected after 12 months off‐treatment, 5‐year survival after relapse was 100% for both groups.^26^ Another study reported 5‐year survival after relapse in those detected by surveillance imaging 64.6% ± 10.1% vs clinical signs and symptoms 73.8% ± 7.2% (*P* = .186).^23^

#### 3.3.2 Osteosarcoma

Five studies of three cohorts reported on 247 patients with osteosarcoma.^28^, ^29^, ^30^, ^31^, ^32^ Where reported, the number of scans during surveillance programs was large, 2394 for 231 patients.^28^, ^31^

Forty‐three patients experienced relapse, with one study providing comparative data on the numbers of patients who experienced relapse detected by surveillance imaging vs symptoms, 7/28 vs 21/28, respectively.^28^ Survival data were largely lacking. Korholz et al reported a 5‐year overall survival of 67%, without comparative data on the method of relapse detection.^28^

#### 3.3.3 Ewing’s sarcoma

Four studies of three cohorts of 355 patients with Ewing’s sarcoma were reported.^30^, ^33^, ^34^, ^35^ About 181 patients relapsed, 87 (48.0%) of which were detected by surveillance imaging and 94 (52.0%) by clinical signs and symptoms.

One study reported a shorter median time to relapse, 0.28 vs 1.22 years, and a lower 5‐year survival, 0% vs 17%, in symptomatic patients compared to those detected by surveillance imaging.^33^ Another study also reported a shorter nonsignificant median time to relapse, 1.6 vs 1.9 years (*P* = .07), between symptomatic and surveillance detection.^34^ Another study found that 5‐year overall survival (OS) after relapse was higher in asymptomatic patients vs symptomatic patients, 37% vs 9%.^35^

#### 3.3.4 Wilms tumor

Six studies explored five cohorts of 5074 patients with Wilms tumor.^17^, ^36^, ^37^, ^38^, ^39^, ^40^ These experienced 836 relapses; where method of relapse detection was reported, 501 were detected by surveillance imaging and 181 detected by symptoms. Not all patients had method of relapse detection reported.

Three of the cohorts contributed few data, with < 20 relapses per study.^37^, ^38^, ^39^ From the two larger cohorts, 5‐year OS after relapse was 56% and 67%.^36^, ^40^ Overall survival for those detected by surveillance vs symptoms was 70% (95% CI, 63% to 77%) vs 59% (95% CI, 46% to 72%).^40^ One study reported a lower median duration of survival after relapse for patients detected by symptoms (22 months with symptoms, not reached for asymptomatic, hazard ratio 1.84 (95% CI 1.24‐2.74)).^36^

Other relevant outcomes included the number of scans to detect one subclinical relapse of 112 (95% CI 106‐119) during the first 2 years after nephrectomy and 500 (95% CI 416‐588) 2‐5 years after nephrectomy and a cost for follow‐up studies of $347,968 per patient.^36^, ^37^

#### 3.3.5 Hepatoblastoma

There were 73 patients with hepatoblastoma included in three studies.^17^, ^41^, ^42^ In two studies, 5/42 patients relapsed, all detected by rise in alpha‐fetoprotein (AFP) levels rather than imaging.^17^, ^42^ One study did not report the number of patients relapsed but stated that all were detected by rise in AFP levels prior to imaging.^41^

In total, 408 imaging studies were performed, although two studies only reported CT data without other imaging types. No study reported data on overall survival. One study found no significant difference in time to relapse between patients detected by surveillance and those detected by symptoms.^42^

#### 3.3.6 Neuroblastoma

Five studies described 487 patients with neuroblastoma, mostly high‐risk disease, of whom 272 relapsed.^43^, ^44^, ^45^, ^46^, ^47^ Where reported, 149 were detected by surveillance imaging and 82 by symptoms.

Survival statistics were variably reported, with most studies reporting less than 5‐year follow‐up. Few patients survived following relapse (n = 2 in CR, three alive with disease of 28 relapses).^43^, ^44^, ^46^ Curve‐estimated 5‐year overall survival after relapse is around 3% in those detected by surveillance and 0% in those detected by symptoms.^45^

One study reported a mean of 29.5 CT scans per patient and another reported a median of 35 images (median CED 133.5 mSv) from the time of initial diagnosis to relapse.^44^, ^47^

#### 3.3.7 Retinoblastoma

Two studies assessed 65 patients with retinoblastoma.^48^, ^49^ Three patients relapsed, one was detected by surveillance imaging and two by symptoms. A total of 223 scans were conducted and 11 false positive images were reported. Survival data are lacking.

#### 3.3.8 Soft tissue sarcomas

Four studies examined rhabdomyosarcoma and included 466 patients with 325 relapses.^3^, ^50^, ^51^, ^52^ One study included all pediatric soft tissue sarcomas—235 patients with relapsed disease, of whom 150 had rhabdomyosarcoma.^16^

In the studies that only included patients with rhabdomyosarcoma, where method of detection was reported, 85 relapses were detected by surveillance imaging and 140 by symptoms. In Dantonello et al, 90 were detected by surveillance, and 139 by symptoms. One study reported 507 scans in 40 patients, with scanning frequency data not provided by other studies.^50^

Two studies reported survival data.^3^, ^51^ Neither found a significant difference in overall survival between those detected by surveillance and by symptoms. The survival rate was lower in one study compared to the other (surveillance vs symptoms: 20% vs 11% 3‐year survival and 43.3% vs 44.6% 5‐year survival, respectively), as the former included progression of disease along with relapse. ^3^, ^51^

In Dantonello et al, 5‐year overall survival from primary surgery was 40% for those detected by surveillance and 29% for those detected by symptoms.^16^ However, by 10 years, survival was 21% for surveillance and 23% for symptomatic. These differences may reflect different biology of disease being detected by surveillance, with these patients surviving longer.

#### 3.3.9 Other tumors

Eight studies examined 583 patients with unique pediatric malignancies, see Table 1 for details.^53^, ^54^, ^55^, ^56^, ^57^, ^58^, ^59^, ^60^ These studies were generally small, all including less than 70 patients, except for Geldart et al who included 329 patients. No study reported more than 20 relapses. Overall survival was reported in four studies.^53^, ^56^, ^57^, ^58^ No study reported differences in survival between groups based on method of relapse detection.

## 4 DISCUSSION

Evidence on the use of surveillance imaging in pediatric extracranial solid tumors is derived exclusively from observational studies. Surveillance strategies are often poorly reported and variable in design, making replication of many studies impossible. The risk of bias for most studies is significant. Evidence gaps were present in all malignancies and the quality of studies was generally low, with particular issues around confounding and lead‐time bias. Conclusive statements regarding the survival benefit of surveillance imaging cannot be made based on information identified in this review.

We recognize that reporting combinations of different imaging types together makes it challenging to separate out the roles of each modality for different malignancies. Sadly, much of the available literature includes all imaging types and presenting separate findings is currently impossible.

Notwithstanding these limitations, it is possible to establish that surveillance imaging programs result in large number of additional imaging investigations, often associated with notable radiation doses. There is a risk of false positive images, including incidental or uncertain findings, which was particularly present in studies of lymphoma. These may be associated with additional distress for patients and families, as well as further investigations. Even with large numbers of tests, surveillance imaging detected only 57% of relapses identified.

Survival outcomes were generally poorly reported. For most malignancies studied, the data available suggested no significant difference in survival between patients whose relapse was detected by surveillance and those whose relapse was detected by symptoms. One exception to this finding is within Wilms tumor, where detection by surveillance imaging does appear consistent with increased survival. However, the number needed to scan is large and the financial costs of surveillance imaging are high. Summary information with the clinical bottom line for each cancer type is provided in Box 2.

Box 2 **Clinical bottom lines**

*Lymphomas*

. Large numbers of scans and false positive imaging is demonstrated in the literature. More research is needed on whether surveillance imaging provides survival benefit.

*Osteosarcoma*

Large numbers of scans are conducted. A lack of comparative survival data between relapses detected by surveillance vs. symptoms. More research is needed on whether surveillance imaging provides survival benefit.

*Ewing’s sarcoma*

Surveillance imaging may not detect relapse prior to symptoms. Those detected earlier by symptoms may have more aggressive disease and therefore have a lower survival after relapse. Research using appropriate effect measures is needed to infer a survival benefit.

*Wilm’s tumour*

Most relapses were detected by surveillance imaging and this appears consistent with increased survival. Data on survival benefit was reported post relapse and at risk of lead‐time bias, thus should be interpreted with caution. The number needed to scan is large and the financial costs are high.

*Hepatoblastoma*

Tumour markers detected all relapses in the literature prior to surveillance imaging, though there were few relapses reported. Patients received a large number of imaging studies. There is no evidence on the effect of surveillance imaging on survival.

*Neuroblastoma*

Evidence was derived mostly from high‐risk patients. The risk of relapse was high and few patients with relapse survived, regardless of the method of detection. Surveillance programmes involved a large number of scans and a significant radiation dose.

*Retinoblastoma*

Large numbers of scans were conducted and were associated with false positive images. There is no evidence on the effect of surveillance imaging on the time to detection of relapse or on survival.

*Soft tissue sarcoma*

Numbers of scans were high and most relapses were detected by symptoms. Evidence does not support improved survival after relapse of rhabdomyosarcoma in those detected by surveillance imaging. For patients with other soft tissue sarcomas evidence is inconclusive.

*Other tumours*

Minimal data is available on the impact of surveillance in rarer diseases and no evidence suggests improved survival with surveillance.

It is important to recognize that any differences in survival reported in these nonrandomized studies may be due to the variable biology of relapsed disease rather than an effect of surveillance. As such, randomized studies are necessary in order to truly evaluate the role of routine surveillance imaging in pediatric patients with extracranial solid tumors.

No qualitative data, psychological distress indicator studies, or studies exploring morbidity or burden of relapse treatment, including the risk of secondary malignancies, were identified. As such, the literature captures little of the patient’s or family’s experience of routine surveillance programs, which may be positive, negative, or both, or of the subsequent treatment of relapse. This is particularly disappointing given our PPI group stressed the importance of these issues. They highlighted to us the importance of understanding the “sawtooth” of anxiety relating to scanning (“scanxiety”), where the anxiety builds to the point of receiving the results of a scan, followed by the relief of a result showing no evidence of disease. They discussed that there may be different anxieties experienced if routine surveillance imaging was not undertaken. They also felt the literature should reflect that knowing about a relapse in advance may not change survival but may alter how life was lived, and thus a deeper exploration of the meanings surrounding surveillance imaging would be a key contribution to the literature in the future. We strongly recommend that high‐quality qualitative research should be performed to understand the various roles of follow‐up, the meaning assigned to surveillance imaging, and the preferences of patients, parents, and professionals in this setting. This research should include both those undergoing routine disease surveillance and those who are not.

The strengths of this review lie in the robust systematic review methodology, informed by extensive PPI engagement focused on design, interpretation, and dissemination. One key challenge lies in how to address teenage and young adult malignancies in systematic reviews. We excluded studies where the majority of participants were over 25. For some diseases, where the population prevalence straddles this cutoff (eg, Hodgkin’s lymphoma and germ cell tumors), this review does not provide all relevant data. Future reviews of these particular malignancies should focus on surveillance across the population.

In addition to this, it is important to recognize that there are challenges in identifying whether patients are symptomatic at the time of surveillance imaging, particularly in retrospective studies. Even if this was identified, this was rarely reported within the literature. We recognize that some patients may have presented with symptoms at the point of surveillance and may have therefore been classified either as symptomatic or detected by surveillance. The effects of this within the data are difficult to predict. It is possible that studies could have consistently classified these patients as one mode of detection over the other. If, for example, patients were more frequently classified as detected by surveillance, it may appear that surveillance images identify more relapses than would be the case in practice. However, it is unlikely to change the duration of survival findings if patients are symptomatic at surveillance visits. Future prospective studies should aim to capture this information so as to inform our understanding of the role of surveillance imaging in this setting.

Concerted effort is required to improve understanding of the risks and benefits of surveillance imaging. We strongly recommend the review of currently held data from research trials or cohorts not currently in the public domain. This may include combining data from multiple studies to inform the research problem. We also recommend the national and international trial bodies to consider including the randomization of follow‐up policies within future trial platforms so as to provide further information about best surveillance practices. Furthermore, we recognize that surveillance imaging programs should change over time, as both up‐front and relapse therapies change, and as the imaging modalities available for surveillance develop, resulting in a different balance of risks and benefits for patients.

## 5 CONCLUSIONS

At present, there is insufficient evidence to evaluate the effects of routine surveillance imaging on survival in most pediatric extracranial solid tumors. More high‐quality focused research is needed that uses appropriate effect measures to address the research questions, alongside well‐conducted qualitative data. This should be a key research priority considering the substantial impact of imaging on patient experience, and the financial and opportunity costs to health services.

Funding: This study was funded by a Children’s Cancer and Leukaemia Group (CCLG) 40^th^ Anniversary Grant. JEM is supported by an NIHR Clinical Lectureship and RSP by an NIHR Postdoctoral Fellowship. Neither NIHR nor CCLG have had any role in the design and conduct of the study; collection, management, analysis, and interpretation of the data; preparation, review, or approval of the manuscript; and decision to submit the manuscript for publication.

## CONFLICTS OF INTEREST

JEM is supported by an NIHR Clinical Lectureship and RSP by an NIHR Postdoctoral Fellowship.

## AUTHOR CONTRIBUTIONS

JEM and RSP conceived the study idea and obtained funding. JEM, MH, and RSP designed the protocol. MH performed the searches. JEM, RW, and RSP screened, selected, quality assessed, extracted data, and analyzed the study. All authors contributed to and have approved the final manuscript.

## Supporting information

Supplementary Material1

Supplementary Material2

Supplementary Material3

## Data Availability

This study reports a systematic review for which all data are already available within the public realm in the form of scientific publications, references for which are provided.
