# Supplementary figures and images for: A systematic review of evidence for and against routine surveillance imaging after completing treatment for childhood extracranial solid tumors
Source: Cancer Med. 2020 May 19;9(14):4949–61. doi: 10.1002/cam4.3110 (PMC7367646; doi:10.1002/cam4.3110)

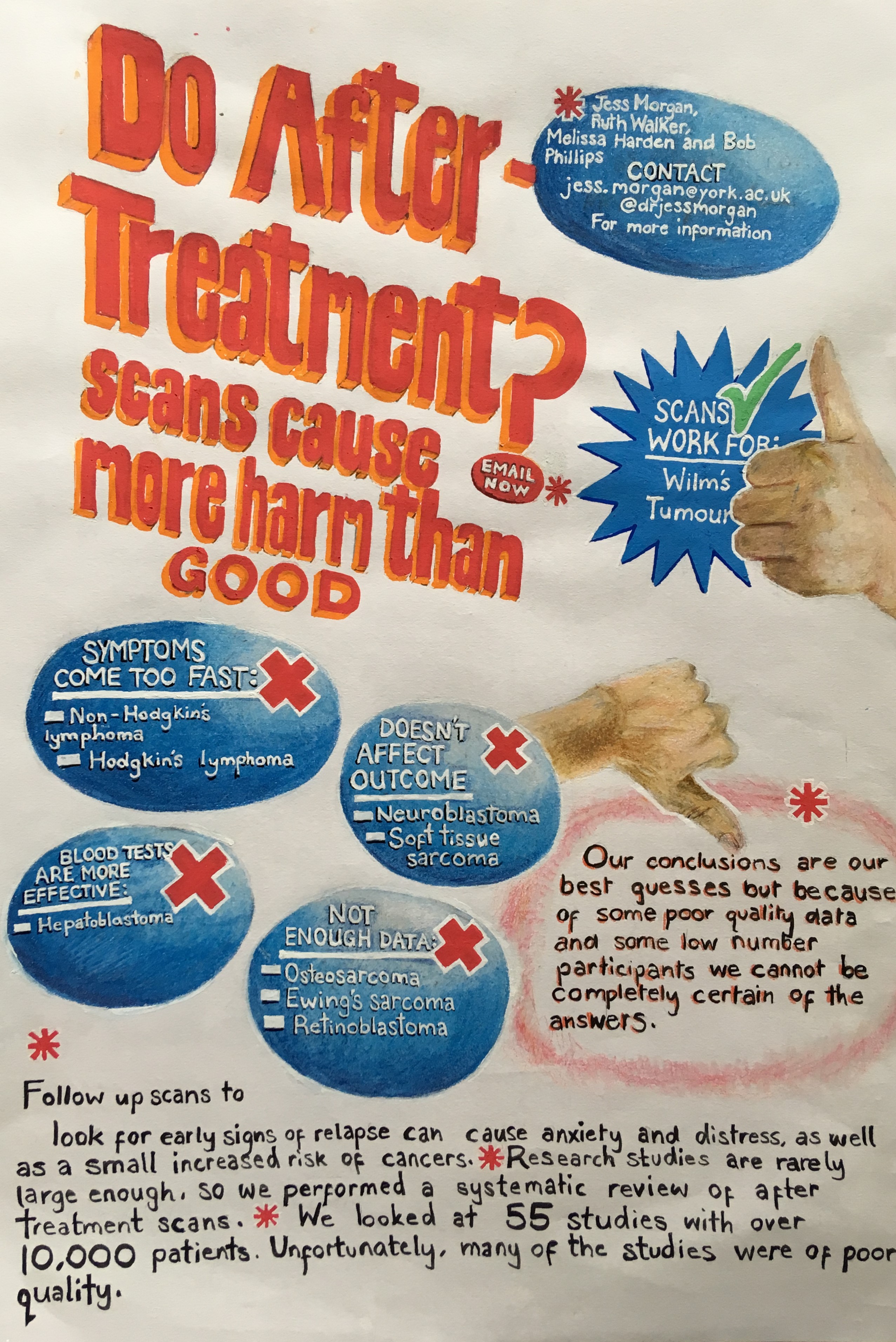

Supplement: Supplementary file 1 — Supplementary Material1 [file CAM4-9-4949-s001.JPG]
