# Supplementary material for: A systematic review of evidence for and against routine surveillance imaging after completing treatment for childhood extracranial solid tumors
Source: Cancer Med. 2020 May 19;9(14):4949–61. doi: 10.1002/cam4.3110 (PMC7367646; doi:10.1002/cam4.3110)
Supplement: Supplementary file 3 — Supplementary Material3 [file CAM4-9-4949-s003.docx]

## Information relating to mixed malignancy studies.

#### Mixed lymphomas

Six studies reported on five mixed cohorts of 176 patients with either Non-Hodgkin’s lymphoma or Hodgkin’s lymphoma.^1–6^

Forty-one patients experienced relapse. Survival data was absent from these studies. Where reported, a total of 226 scans were performed in 108 patients.^1–3^

#### Other mixed malignancy studies

Four mixed malignancy studies reported on 1916 patients, including 248 relapses.^7–10^ One study included some patients with leukaemia (16 of 101 relapses)^8^ another included 27 brain tumour patients^7^ and where possible have been removed from this review. Where reported, 101 relapses were detected by surveillance imaging and 147 detected by symptoms.

One study reported 10 year overall survival after relapse was 20.5% in those diagnosed through scheduled visits and 32.1% for those diagnosed on an unscheduled visit (p=0.83).^8^

Two studies reported 9217 scans on 478 patients.^7,10^ For the 40 rhabdomyosarcoma patients in one study, the surveillance imaging cost was $345,579.79, of which 65% was for surveillance of patients who never relapsed.

Howell et al reported the number of scans needed to detect one relapse; 42 for MRI scans, 129 for CT and 257 for chest radiography.^7^ The cost per recurrence for all malignancies was over £1900, other than nasopharyngeal carcinoma (£897).

#### Clinical bottom line

Due to clinical heterogeneity the effect of surveillance imaging in mixed malignancy studies is unclear. Most relapses were detected due to symptoms, and surveillance imaging involves high numbers of images as well as financial costs.

### References

1. Csoka M, Bardy E, Nemes K, Szegedi I, Kiss C, Kovacs G. The role of FDG-PET/CT in follow-up of children with lymphoma. *Pediatr Blood Cancer*. 2010;55 (5):870.

2. Depas G, De Barsy, C, et al. 18F-FDG PET in children with lymphomas. *Eur J Nucl Med Mol Imaging*. 2005;32(1):31-8.

3. Lopci E, Burnelli R, Ambrosini V, et al. (18)F-FDG PET in Pediatric Lymphomas: A Comparison with Conventional Imaging. *Cancer Biother Radiopharm*. 2008;23(6):681-90.

4. Bueno JL, Abdeen N. Imaging followup of lymphoma in pediatric patients: Is pelvic CT necessary? *Pediatr Radiol*. 2012;2):S307.

5. Brisse H, Pacquement H, Burdairon E, Plancher C, Neuenschwander S. Outcome of residual mediastinal masses of thoracic lymphomas in children: impact on management and radiological follow-up strategy. *Pediatr Radiol*. 1998;28(6):444-50.

6. Csoka M, Bardi E, Nemes K, Szatmary B, Muller J, Kovacs G. The role of FDG-PET/CT in follow-up of children of children with lymphoma and osteosarcoma. *Haematol Meet Rep*. 2009;3 (5):92.

7. Howell L, Mensah A, Brennan B, Makin G. Detection of recurrence in childhood solid tumors. *Cancer*. 2005;103(6):1274-1279.

8. Biasotti S, Garaventa A, Padovani P, et al. Role of active follow-up for early diagnosis of relapse after elective end of therapies. *Pediatr Blood Cancer*. 2005;45(6):781-6.

9. Butala A, Woodman J, Rademaker A, et al. Recurrence detection in children with extra-cranial tumors at Ann & Robert H. Lurie Children’s Hospital (LCH) of Chicago. *J Clin Oncol Conf*. 2015;33(15 SUPPL. 1).

10. Zimmerli J, Maese L, Muir S, Korgenski K, Schiffman J. A retrospective study evaluating the utility of off-therapy surveillance imaging of non-CNS solid tumors with a focus on rhabdomyosarcoma. *Pediatr Blood Cancer*. 2017;64 (Supplement 1):S75.

11. Dantonello TM, Winkler P, Kube S, et al. Do children, adolescents, and young adults with soft tissue sarcoma benefit from early detection of recurrences? Results of a population-based study. *J Clin Oncol Conf*. 2012;30(15 SUPPL. 1).

12. Kan JH, Hwang M, Lowas SR, Hernanz-Schulman M. Impact of pelvic CT on staging, surveillance, and survival of pediatric patients with Wilms tumor and hepatoblastoma. *AJR Am J Roentgenol*. 2011;196(5):W515-8.

Table 1. Study characteristics characteristics of mixed malignancy studies:

| **Study** | **Year of publication** | **Disease** | **Country** | **N participants** | **N participants experiencing relapse** | **Age (median ¹, mean ²)** | **Details of surveillance programme** | | |
| --- | --- | --- | --- | --- | --- | --- | --- | --- | --- |
|  |  |  |  |  |  |  | **Mode** | **Frequency** | **Maximum follow up period** |
| Brisse^5^ | 1998 | Lymphomas (mixed) | France | 39 | 1 | 11² | CT, X-ray | 2-3 times per year | 10 |
| Bueno^4^ | 2012 | Lymphomas (mixed) | Canada | 29 | 3 | 11.9² | CT | NR | NR |
| Csoka^6^ | 2009 | Lymphomas (mixed) | Hungary | 30 | 18 | 13.7¹ | CT, FDG-PET | NR | NR |
| Csoka^1^ | 2010 | Lymphomas (mixed) | Hungary | 60 | 35 | 13.5¹ | CT, FDG-PET | NR | 9 |
| Depas^2^ | 2005 | Lymphomas (mixed) | Belgium | 28 | 0 | 12.5² | FDG-PET | NR | 9.25 |
| Lopci^3^ | 2008 | Lymphomas (mixed) | Italy | 20 | 2 | 10² | CT, Ultrasound, X-ray, MRI, FDG-PET, Bone scan | NR | 3 |
| Biasotti^8^ | 2005 | Mixed malignancies | Italy | 739 | 101 | 4.9² | NR | NR | 16 |
| Butala^9^ | 2015 | Mixed malignancies | US | 699 | 67 | NR | NR | NR | 5 |
| Dantonello^11^ | 2012 | Mixed malignancies | Germany | 235 | 229 | NR | NR | NR | NR |
| Howell^7^ | 2005 | Mixed malignancies | UK | 186 | 23 | 5.4² | CT, Ultrasound, X-ray, MRI, FDG-PET, Bone scan | NR | 10.5 |
| Kan^12^ | 2011 | Mixed malignancies | US | 55 | 3 | 2.4² | CT | NR | 12.08 |
| Zimmerli^10^ | 2017 | Mixed malignancies | US | 63 | 57 | NR | NR | NR | NR |

Table 2. Risk of bias for mixed malignancy studies

| **Study** | **Year** | **Confounding** | **Patient selection** | **Protocol deviation** | **Missing data** | **Knowledge of intervention and recording of outcome** | **Effect estimate** | **Overall judgement of risk** |
| --- | --- | --- | --- | --- | --- | --- | --- | --- |
| Biasotti^8^ | 2005 |  |  |  |  |  |  | Serious |
| Brisse^5^ | 1998 |  |  |  |  |  |  | Moderate |
| Bueno^4^ | 2012 |  |  |  |  |  |  | Moderate |
| Butala^9^ | 2015 |  |  |  |  |  |  | Moderate |
| Csoka^6^ | 2009 |  |  |  |  |  |  | Moderate |
| Csoka^1^ | 2010 |  |  |  |  |  |  | Moderate |
| Dantonello^11^ | 2012 |  |  |  |  |  |  | Moderate |
| Depas^2^ | 2005 |  |  |  |  |  |  | Moderate |
| Howell^7^ | 2005 |  |  |  |  |  |  | Moderate |
| Kan^12^ | 2011 |  |  |  |  |  |  | Moderate |
| Lopci^3^ | 2008 |  |  |  |  |  |  | Moderate |
| Zimmerli^10^ | 2017 |  |  |  |  |  |  | Moderate |

Key: Blue: no information; Green: low risk; Orange: moderate risk; Red: serious risk; Purple: critical risk.
